# Turmeric rhizomes reduced *in vitro* methane production and improved gas production and nutrient degradability

**DOI:** 10.1080/10495398.2024.2371519

**Published:** 2024-07-11

**Authors:** Ahmed E. Kholif, Olurotimi A. Olafadehan, Gouda A. Gouda, Mahmoud Fahmy, Tarek A. Morsy, Hajer Ammar, Hatem A. Hamdon, Mireille Chahine

**Affiliations:** a Dairy Science Department, National Research Centre, Giza, Egypt; b Department of Animal Sciences, North Carolina Agricultural and Technical State University, Greensboro, NC, USA; c Department of Animal Science, University of Abuja, Abuja, Nigeria; d High Agriculture School of Mograne, University of Carthage, Tunisia; e Department of Animal Production, Faculty of Agriculture, New Valley University, New Valley, Egypt; f Department of Animal, Veterinary and Food Sciences, University of Idaho, Twin Falls, ID, USA

**Keywords:** Degradability, *in vitro* fermentation, methane, phytogenics, turmeric

## Abstract

The present study aimed to evaluate the effect of dry turmeric rhizomes on *in vitro* biogas production and diet fermentability. Turmeric rhizomes were included at gradually increased levels: 0, 0.5, 1, 1.5 and 2% of a diet containing per kg dr matter (DM): 500 g concentrate feed mixture, 400 g berseem hay and 100 g rice straw, and incubated for 48 h. Gas chromatography-mass spectrometry analysis showed that *ar*-turmerone, *α*-turmerone and *β*-turmerone were the major bioactive compounds in the rhizomes. Turmeric rhizomes increased (*p* < 0.01) asymptotic gas production (GP) and rate and lag of CH_4_ production and decreased (*p* < 0.01) rate of GP, lag of GP, asymptotic CH_4_ production and proportion of CH_4_ production. Turmeric rhizome administration linearly increased (*p* < 0.01) DM and fiber degradability and concentrations of total short-chain fatty acids, acetic and propionic acids and ammonia-N and quadratically (*p* < 0.05) decreased fermentation pH. It is concluded that including up to 2% turmeric rhizomes improved *in vitro* ruminal fermentation and decreased CH_4_ production.

## 1. Introduction

Animal nutritionists and microbiologists seek to improve feed utilization, ruminal fermentation and animal performance and reduce methane (CH_4_) emission. Feed additives from chemical or organic sources showed mixed effects on animal performance. However, those from organic/natural sources are recommended as potential alternatives to antibiotics due to concerns regarding antibiotic resistance, drug residues and consumer health.^1^ Therefore, there is an increasing global focus on natural, green and multifunctional feed additive products as alternatives to antibiotics. Phytogenic feed additives are organic feed additives that have gained increasing interest from livestock producers. They are rich in bioactive secondary metabolites that alter ruminal fermentation and affect animal performance.^2–4^ The phytoconstituents of herbs and medicinal plants significantly affect ruminal microorganism activity in different ways depending on their dose, source and type.^5–7^ However, the dose of the phytoconstituents is a premium issue in determining the effectiveness of these substances as feed additives to improve ruminal fermentation and digestion.^8^^,^^9^

Phytochemicals showed positive effects on reducing CH_4_ emissions from ruminants,^5^^,^^10^ due to their effects on ruminal methanogens and methanogenesis.^5^^,^^6^^,^^11^ They mitigate ruminal CH_4_ production by directly inhibiting ruminal methanogenic archaea and/or suppressing microbial metabolic processes involved in methanogenesis.^6^ Phytochemicals have high antimicrobial activity against most ruminal microorganisms, especially methanogens, due to their contents of terpenoids, phenolics and phenols.^5^^,^^6^^,^^12^ However, the mode of action of reducing CH_4_ production differs between phytochemicals. Moreover, phytochemicals alter ruminal archaea and reduce methanogenic activity.^6^^,^^12^^,^^13^

Turmeric rhizomes are rich in plant polyphenols, which showed numerous effects on short-chain fatty acids (SCFA) of ruminants,^14^ nitrogen (N) utilization^15^ and the activity and population of rumen microorganisms.^5^ Turmerone and curcumin are the primary compounds in the rhizomes of turmeric.^16^ Tian et al.^16^ showed that the inclusion of curcumin in the diet of lambs at 300, 600 or 900 mg/kg diet increased average daily gain without affecting intake or feed conversion. Moreover, curcumin feeding at a 300 mg/kg diet increased total SCFA and acetate, while it decreased ruminal pH and ammonia-N (NH_3_-N) concentration. Aderinboye and Olanipekun^17^ stated that turmeric inclusion above 10 mg/g DM was detrimental to ruminal fermentation and caused negative effects on fermentation end products and microbial biomass synthesis. Therefore, the objectives of the present experiment were to evaluate the effect of the inclusion of dried turmeric rhizomes at different levels in a total mixed ratio (TMR) on *in vitro* gas production (GP), CH_4_ production and *in vitro* ruminal fermentation. We hypothesized that the phytochemicals in the dried turmeric rhizomes would affect the ruminal microorganisms, alter ruminal fermentation and improve nutrient degradability.

## 2. Materials and methods

### 2.1. Ingredients and treatments

A TMR used as substrate was prepared to contain per kg dry matter (DM): 500 g concentrate feed mixture (CFM), 400 g berseem hay and 100 g rice straw. The CFM contained per kg DM: 170 g soybean seed meal, 395 g wheat bran, 395 g maize, 20 g limestone, 10 g vitamins and minerals mixture and 10 g salt. Nutrient contents of turmeric, ingredients and TMR are shown in Table 1.

**Table 1.** Chemical composition of turmeric and incubated diet (g/kg DM).

	Turmeric	CFM^a^	Berseem hay	Rice straw	Diet^b^
Dry matter	917	903	890	940	893
Organic matter	929	923	884	851	819
Crude protein	59	165	128	42	136
Ether extract	79	47	54	19	62
Nonstructural carbohydrates	650	414	224	166	359
Neutral detergent fiber	141	297	478	624	379
Acid detergent fiber	663	175	381	394	240
Gross energy^c^	4.40	4.35	4.17	3.73	3.95
Digestible energy^c^	4.26	4.22	4.05	3.62	3.83
Metabolizable energy^c^	3.88	3.82	3.65	3.20	3.44
Net energy for lactation^c^	2.54	2.50	2.38	2.06	2.23

^a^Concentrated feed mixture (CFM) contained per kg DM: 170 g soybean meal, 395 g wheat bran, 395 g maize, 20 g limestone, 10 g vitamins, minerals mixture and 10 g salt.

^b^Diets: contained per kg DM: 500 g concentrate mixture, 400 g berseem hay and 100 g rice straw.

^c^Calculated according to the equations of the NRC^23^ as gross energy (GE, Mcal/kg DM) = CP × 0.056 + EE × 0.094 + carbohydrate × 0.042.

Digestible energy (DE, Mcal/kg DM) = 0.97 × GE.

Metabolizable energy (ME, Mcal/kg DM) = (1.01 × DE − 0.45) + 0.0046 (EE − 3).

Net energy for lactation (NEL, Mcal/kg DM) = 0.703 × ME − 0.19.

Clean and dry turmeric rhizomes were obtained from a local supplier in Egypt. Rhizomes were grounded, through a 1-mm screen using a Wiley mill (Arthur H. Thomas, Philadelphia, PA, USA), and mixed before use. Gas chromatography-mass spectrometry (GC-MS) was used to measure essential oils in the rhizomes at the Central Laboratory of National Research Center (Egypt) using a Perkin Elmer Auto System XL GC-MS (Agilent, USA), and a capillary column ZB-5 (60 m × 0.32 mm i.d.; Agilent, USA). The analysis was done according to Qin et al.^18^ with some modifications. The injector temperature was set to 50 °C for 1 min, then programmed to 240 °C at 3 °C/min. Helium was used as the carrier gas at the rate of 1 mL/min with a split vent flow of 1:10. The effluent at the GC column was introduced directly to the source of the MS. Spectra were obtained in the EI mode with 70 eV ionization energy. The sector mass analyzer was set to scan from 40 to 300 amu for 1 s. A tentative identification of the compounds was performed based on the comparison of their relative retention time and mass spectra with those of the NIST and WILLY library data of the GC-MS system.

### 2.2. In vitro fermentation and biodegradation

The *in vitro* fermentation medium was prepared according to Goering and Van Soest.^19^ A reducing solution containing sodium sulfide was added (2 mL) to the buffer shortly before the rumen fluid addition. Ruminal inoculum (20 mL) and the buffer solution (80 mL) were mixed in each 250 mL bottle. Three separate incubation runs were performed using rumen liquor from 3 animals for each run (3 runs).

For each run, ruminal inoculum was collected from the rumen of three slaughtered Barki sheep from a local slaughterhouse in Cairo (Egypt). Sheep were slaughtered according to the Egyptian and Islamic laws of animal slaughtering and carcass handling, where any stress or discomfort to the animals was avoided and animals were handled gently with care. Before slaughtering, sheep were *ad libitum* fed a diet containing concentrate (200 g soybean seed meal, 400 g wheat bran, 360 g maize, 20 g limestone, 10 g vitamins and minerals mixture and 10 g salt), berseem hay and rice straw at 500:400:100 (DM basis), with free access to water. The ruminal fluid was filtered through two-layered cheesecloth to remove large feed particles and the particulate materials were squeezed to obtain microbes attached to feed particles. The initial pH of the inoculum was varied from 6.8 to 6.9. All replacement levels were tested in 3 incubation runs with three replicates (bottles; analytical replicates) in each run. In each incubation run, two bottles with inoculum but without feed (blanks) were also included to establish baseline fermentation GP.

A 1 g ± 10 mg sample of the formulated TMR was weighed into filter bags (ANKOM F57; Ankom Technology, Macedon, NY, USA) and then placed into 250 mL ANKOM bottles (Ankom^RF^ Gas Production System) fitted with an automatic wireless *in vitro* GP module (Ankom Technology, Macedon, NY, USA) with pressure sensors. Turmeric rhizomes were included at 0 (control-no additives), 0.5, 1, 1.5 and 2% of the diet. The pressure was recorded every 10 min for 48 h, and cumulative pressure was calculated from these values. The gas pressure was converted into volume (mL) at standard pressure and temperature. The gas volume in the blank units was subtracted to obtain net GP. At 2, 4, 6, 8, 10, 12, 24, 36 and 48 h incubation time, gas samples (5 mL) were taken from the sampling vent and infused into a Gas-Pro detector (Gas Analyzer CROWCON Model Tetra3, Abingdon, UK) to measure the concentration of CH_4_.

### 2.3. Sampling and analysis of fermentation variables

After 48 h of incubation, fermentation was stopped by placing the bottles on ice for 5 min, and the pH was measured immediately using a pH meter. The ANKOM F57 filter bags were dried in a forced air oven at 55 °C for 48 h. Dry matter, neutral detergent fiber (NDF) and acid detergent fiber (ADF) degradation were calculated by subtracting the dried residue weight from the initial weight of the dried substrate. Total gas and CH_4_ productions were expressed per gram of degraded DM, NDF and ADF at 48 h of incubation.

Samples (5 mL) of the supernatant fermented fluid from each bottle were collected in glass tubes to determine NH_3_-N and total and individual SCFA concentrations. A subsample of 3 mL was preserved with 3 mL of 0.2 M hydrochloric acid solution for the measurement of NH_3_-N concentration using Kjeldahl digestion according to AOAC^20^ (method ID 954.01). Samples (100–200 mg) in 5 mL of concentrated H_2_SO_4_ for 1.5 h in a micro Kjeldahl digestion unit, where the resulting solutions were made up to 50 mL and were used for the subsequent titrimetric NH_3_-N determination. Steam distillation after Kjeldahl digestion was carried out, and the distillate was collected in an Erlenmeyer flask containing 50 mL of 4% boric acid and Tashiro indicator. Titration was performed using 0.1 M HCl.

An aliquot (0.8 mL) was mixed with 0.2 mL of metaphosphoric acid solution (250 g/L) for total and individual SCFA analysis using a high-performance liquid chromatography (HPLC). Analysis was carried out using an Inert Sustain as previously noted by Abdel-Nasser^21^ with some modifications. The separation was carried out using Eclipse AQ-C18 HP column (4.6 mm x 150 mm i.d., 3 μm). The mobile phase consisted of 0.005 N sulfuric acid. The mobile phase was programmed consecutively in a linear gradient for flow rate as follows: 0–4.5 min (0.8 mL/min); 4.5–4.7 min (1 mL/min); 4.7–4.71 min (1 mL/min); 4.71–8.8 (1.2 mL/min); 8.8–9 (1.3 mL/min); 9–23 (1.3 mL/min) and 23–25 (0.8 mL/min). The diode array detector (DAD) was monitored at 210 nm. The injection volume was 5 μL for each of the sample solutions. The column temperature was maintained at 55 °C. A mixture of known concentrations of individual SCFAs was used as an external standard (Sigma Chemie GmbH, Steinheim, Germany) to calibrate the integrator. The analysis was performed at the Chromatography Laboratory, Central Laboratories Network, National Research Center, Egypt.

### 2.4. Chemical analysis

Samples of turmeric rhizomes, ingredients and TMR were analyzed for ash after burning the samples in a muffle furnace at 550 °C for 12 h (method ID 942.05), crude protein (CP) using the Kjeldahl method (method ID 954.01), and ether extract (EE) using diethyl ether in Soxhlet extractors (method ID 920.39) according to AOAC^20^ methods. Neutral detergent fiber content was determined without alpha amylase but with sodium sulfite, following the procedure of Van Soest et al.^22^ Acid detergent fiber content was analyzed according to AOAC^20^ (method ID 973.18) and expressed exclusive of residual ash. Nonstructural carbohydrate, cellulose, hemicellulose and organic matter (OM) concentrations were calculated. Energy contents, including gross energy (GE), digestible energy (DE), metabolizable energy (ME) and net energy for lactation (NEL), were estimated according to the equations of NRC.^23^

### 2.5. Calculations and statistical analyses

For the estimation of GP and CH_4_ kinetics, data of total GP and CH_4_ (mL/g DM) were fitted using the NLIN procedure of SAS (Version 9.4, SAS Inst., Inc., Cary, NC) according to France et al.^24^ model as y = *A* × [1 − *e*^−^*^c^*^(^*^t^*^−Lag)^] where *y* is the volume of total GP or CH_4_ production at time *t* (h); *A* is the asymptotic GP or CH_4_ (mL/g DM); *c* is the fractional rate of GP or CH_4_ (/h) and Lag (h) is the discrete lag time before any GP or CH_4_ release.

The partitioning factor at 24 h of incubation (PF_24_ = mg degraded DM/mL gas) was estimated.^25^ The volume of gas produced (mL/200 mg DM) at 24 h incubation (GY_24_) was calculated as GY_24_ = mL GP per gram DM/g apparent degraded substrate (ADS). Metabolizable energy was calculated (2.20 + 0.136 × GP + 0.057 × CP) according to Menke et al.^26^ Microbial crude protein (MCP) production was calculated (MCP = mg ADS-(mL gas × 2.2 mg/mL)) according to Blümmel et al.^25^

Data were analyzed using the GLM procedure of SAS in a complete randomized design using the model: *Y_ij_* = *μ* + *L_i_* + *ε_ij_* where: *Y_ij_* is the observation, *μ* is the population mean, *L_i_* is the turmeric level effect and *ε_ij_* is the residual error. Data from each of the three runs of the same substrate sample were averaged before statistical analysis. The mean values of each run were used as the experimental unit, considering the run effect as a covariate. Linear and quadratic contrasts were employed to determine the level responses (increasing turmeric levels). Comparisons among treatments were performed with Tukey’s range test.

## 3. Results

### 3.1. Turmeric

*ar*-turmerone, *α*-turmerone and *β*-turmerone were the major compounds in the rhizomes of turmeric (Table 2).

**Table 2.** Volatile compounds in turmeric identified by GC-MS analysis.

Compounds^a^	Concentration (%)^b^
*ar*-Turmerone	36.5
*α*-Turmerone	24.2
*β*-Turmerone	17.7
*α*-Phellandrene	6.17
Eucalyptol	3.2
Camphor	2.61
Fenchone	2.27
Terpinolene	1.53
*α*-Curcumene	1.44
Anethole	1.01
*β*-Curcumene	0.82
*α*-Humulene	0.73
*β*-pinene	0.67
1-Chloro-5-methyl hexane	0.64
*α*-pinene	0.51

^a^Identification based on authentic standards, National Institute of Standards and Technology (NIST) library spectra and literature.

^b^Concentration based on the total areas of the identified peaks.

### 3.2. Biogas production

**Figure 1.**
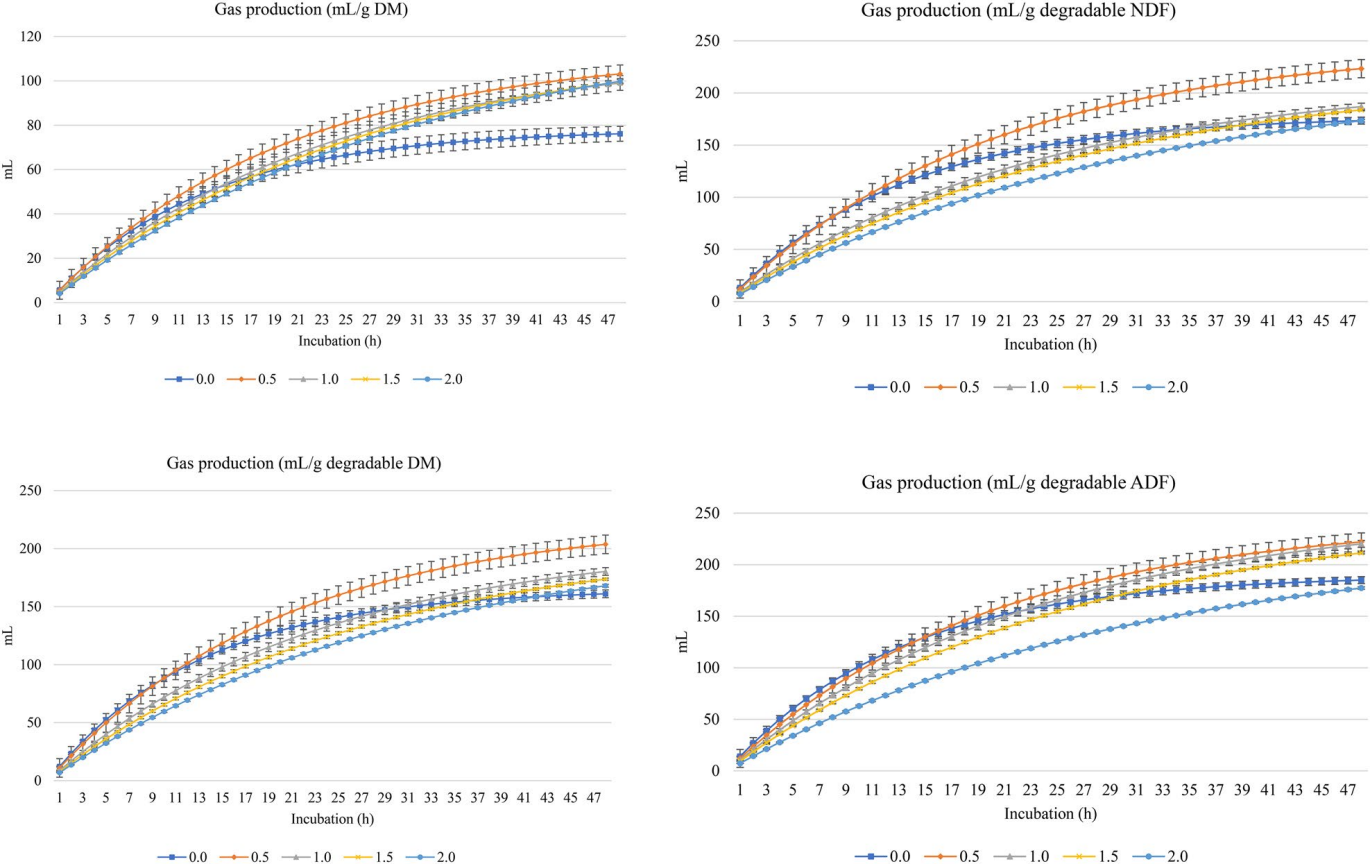
*In vitro* ruminal gas production of a total mixed ration supplemented with turmeric at 0, 0.5, 1, 1.5, or 2% (DM basis). NDF is neutral detergent fiber degradability and ADF is acid detergent fiber.

**Figure 2.**
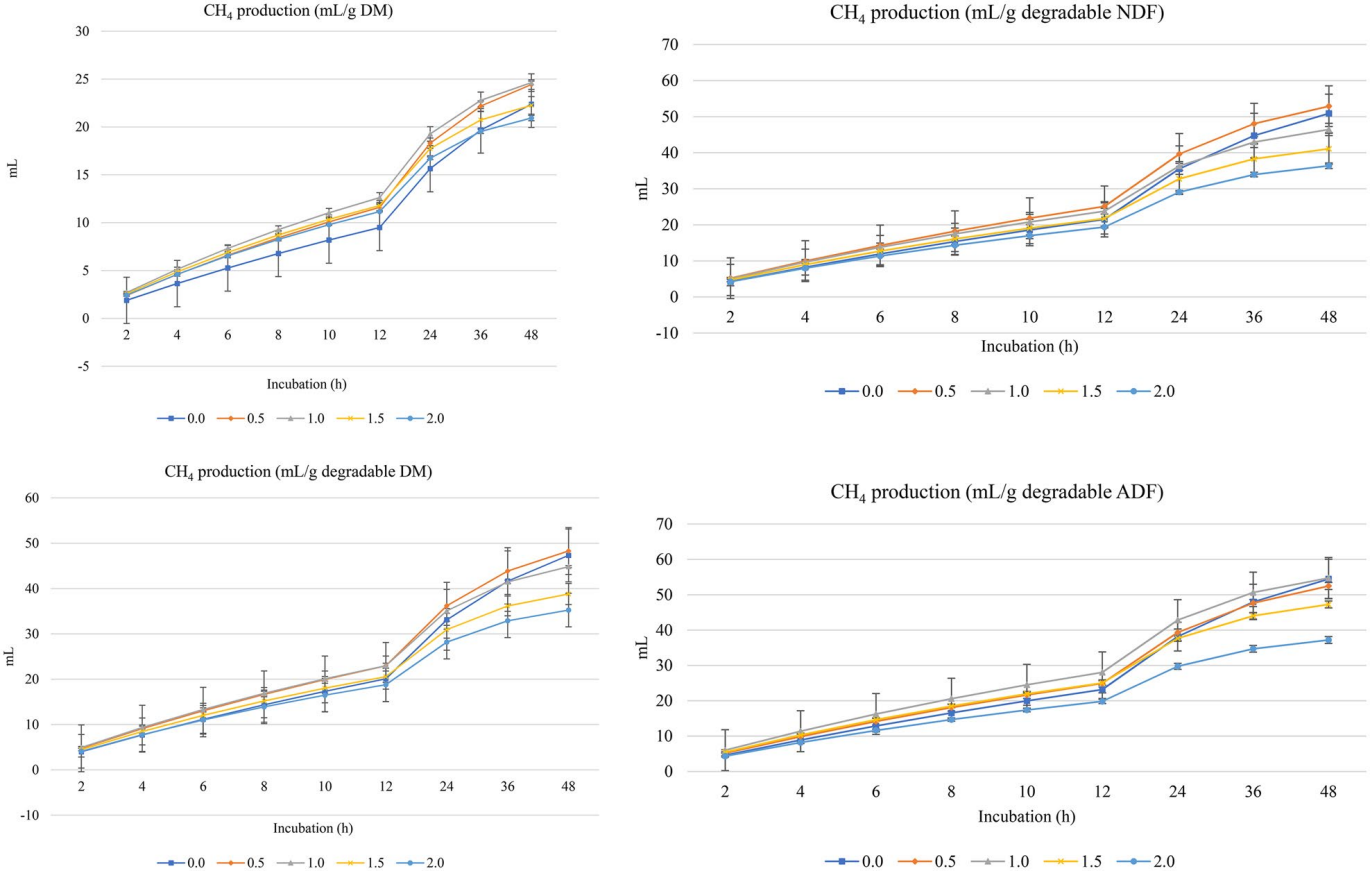
*In vitro* ruminal methane (CH_4_) production of a total mixed ration supplemented with turmeric at 0, 0.5, 1, 1.5, or 2% (DM basis). NDF is neutral detergent fiber degradability and ADF is acid detergent fiber.

Figures 1 and 2 show GP (mL) and CH_4_ (mL), respectively, per g DM, degradable DM, degradable NDF, and degradable ADF. Turmeric rhizomes increased (linear and quadratic effects, *p* < 0.001) the asymptotic GP and decreased the rate of GP (linear and quadratic effects, *p* < 0.001) and the lag of GP (linear and quadratic effects, *p* < 0.05) (Table 3).

**Table 3.** *In vitro* rumen gas production (GP) and methane (CH_4_) kinetics and cumulative gas production after 48 h of incubation as affected by increasing levels of turmeric (%, dry matter basis).

Level	GP parameters^a^			CH_4_ parameters^b^			
	*A*	*c*	Lag	*A*	*c*	Lag	%^c^
0	78	0.076	1.55	28.6	0.035	1.42	29.3
0.5	114	0.050	1.47	27.5	0.046	1.59	23.7
1	114	0.043	1.42	26.7	0.053	1.63	24.9
1.5	119	0.038	1.35	23.8	0.057	1.68	22.3
2	125	0.033	1.27	22.3	0.058	1.73	21.0
SEM	2.0	0.0010	0.070	0.70	0.0032	0.051	0.68
***p* value**							
Treatment	<0.001	<0.001	0.013	0.004	0.002	0.013	<0.001
Linear	<0.001	<0.001	0.011	<0.001	0.002	0.001	<0.001
Quadratic	<0.001	0.003	0.945	0.308	0.063	0.252	0.091

Means in the same row with different superscripts differ, *p* < 0.05. *p*-value is the observed significance level of the *F*-test for treatment; SEM = standard error of the mean.

^a^GP parameters: *A* is the asymptotic GP (ml/g DM), *c* is the rate of GP (/h) and lag is the initial delay before GP begins (h).

^b^Methane (CH_4_) production parameters: *A* is the asymptotic CH_4_ production (ml/g DM), *c* is the rate of CH_4_ production (/h) and lag is the initial delay before CH_4_ production begins (h).

^c^The proportional CH_4_ at the end of incubation (48 h).

Linear decreases in the asymptotic CH_4_ production (*p* < 0.001) and proportion of CH_4_ production (*p* < 0.001), and increased rate of CH_4_ (*p* = 0.002) and lag of CH_4_ production (*p* = 0.001) were observed with turmeric rhizomes inclusion (Table 3).

### 3.3. Degradability and fermentation

Linear increases (*p* < 0.01) in the degradability of DM, NDF and ADF were observed with the inclusion of turmeric rhizomes (Table 4). Moreover, turmeric rhizomes linearly increased (*p* < 0.01) the concentrations of total SCFA, acetic and propionic acids, NH_3_-N, ME, PF_24_ and MCP; however, they quadratically decreased fermentation pH (*p* = 0.023) without affecting butyrate concentration or acetic acid-to-propionic acid ratio.

**Table 4.** *In vitro* rumen fermentation profile of diet with increasing levels of turmeric (%, dry matter basis).

Level	Degradability^a^			SCFA^b^						Fermentation^c^			
	DM	NDF	ADF	Total SCFA	C_2_	C_3_	C_2_:C_3_	C_4_	pH	NH_3_–N	ME	PF_24_	MCP
0	473	439	412	23.4	11.4	7.90	1.47	4.08	6.27	10.43	4.68	7.22	328
0.5	508	462	470	24.3	12.2	7.79	1.56	4.30	6.10	11.27	4.70	6.42	334
1	549	531	450	26.4	12.7	8.69	1.47	4.99	6.10	11.43	4.52	7.55	389
1.5	574	543	472	26.3	13.4	9.21	1.45	3.68	6.17	12.13	4.47	8.08	418
2	594	575	563	27.5	13.6	9.34	1.46	4.56	6.15	13.03	4.41	8.64	443
SEM	11.6	8.3	17.9	0.40	0.34	0.297	0.090	0.559	0.036	0.476	0.056	0.213	11.2
***p* value**													
Treatment	0.001	<0.001	0.002	0.001	0.006	0.011	0.902	0.566	0.044	0.030	0.016	0.003	<0.001
Linear	<0.001	<0.001	0.003	<0.001	0.004	0.001	0.658	0.851	0.157	0.002	0.002	<0.001	<0.001
Quadratic	0.300	0.253	0.139	0.331	0.493	0.937	0.780	0.747	0.023	0.716	0.882	0.024	0.765

Means in the same row with different superscripts differ, *p* < 0.05. *p*-value is the observed significance level of the *F*-test for treatment; SEM = standard error of the mean.

^a^DM is the dry matter degradability (mg/g incubated), OM is the organic matter degradability (mg/g incubated), NDF is the neutral detergent fiber degradability (mg/g incubated), ADF is the acid detergent fiber degradability (mg/g incubated).

^b^SCFA is short chain fatty acids (mmol/L), C_2_ is acetate (mmol/L), C_3_ is propionate (mmol/L), C_4_ is butyrate (mmol/L).

^c^NH_3_-N is ammonia-N (mg/g DM), ME is metabolizable energy (MJ/kg DM), PF_24_ is the partitioning factor at 24 h of incubation (mg degradable DM: mL gas), MCP is microbial CP production (mg/g DM).

## 4. Discussion

### 4.1. Turmeric

*ar*-turmerone, *α*-turmerone and *β*-turmerone in addition to *α*-curcumene and *β*-curcumene are the primary volatile compounds in the rhizomes of turmeric. Gounder and Lingamallu^27^ evaluated the chemical composition of volatile oils of turmeric rhizomes and showed that *ar*-turmerone (21.0–30.3%), *α*-turmerone (26.5–33.5%) and *β*-turmerone (18.9–21.1%) were the major components. Moreover, they observed that the antioxidant capacity was 686 mM of ascorbic acid equivalents per 1 mg of oil, while the IC_50_ value was 3.5 mg of oil/mL. In another experiment, Poudel et al.^28^ observed that *ar*-turmerone (25.5%), *α*-turmerone (24.4%), *β*-turmerone (14.0%), terpinolene (7.2%), β-sesquiphellandrene (5.1%), *α*-zingiberene (4.8%), *β*-caryophyllene (2.9%), *ar*-curcumene (1.6%) and 1,8-cineole (1.3%) were the major volatile components in turmeric rhizomes. Feeding additives, rich in volatile components, causes changes in the rumen microbiome and fermentation kinetics,^6^^,^^29^ due to the antimicrobial effects of phenolic and non-phenolic compounds against ruminal microbiota (fungi, protozoa and bacteria). Patra et al.^30^ observed that fennel decreased carboxymethyl-cellulase and xylanase activities. Collectively, such effects on ruminal microbes produce some changes in ruminal fermentation kinetics, as discussed later.

### 4.2. Gas production

All levels of turmeric rhizomes linearly increased the asymptotic GP, indicating improved ruminal fermentability and degradability of the incubated diet, which may be due to the effect of phytochemicals on ruminal microbes.^31^ The phytochemicals in turmeric rhizomes may have decreased protozoal populations,^3^^,^^16^ increased bacterial and fungal populations and improved microbial activity.^6^ In an *in vitro* experiment, Kholif et al.^29^ observed increased GP with fennel seeds at different levels. Phytogenic feed additives, mainly plant secondary compounds, have been shown to modulate rumen microflora and change rumen fermentation dynamics leading to enhanced ruminal fermentation of incubated substrates.^32^^,^^33^ It is clear that the evaluated levels of turmeric were lower than the level (>10 mg/g DM) stated by Aderinboye and Olanipekun^17^ to be detrimental to ruminal fermentation. Aderinboye and Olanipekun^17^ observed lowered *in vitro* GP with turmeric inclusion. This may be due to the higher levels of turmeric (5 to 15%) in their study compared to those in the present experiment (0.5 to 2%). Hodjatpanah-Montazeri et al.^34^ showed that including turmeric extract at 20, 40 and 80 mg/L of fluid increased GP after 96 h of incubation. The presence of phytochemicals (*ar*-turmerone, *α*-turmerone, *β*-turmerone, etc.) in the rhizomes improved ruminal fermentation.^6^

Turmeric rhizome administration linearly decreased the rate and lag of GP, which confirms the inverse relationship between the asymptotic GP and the rate of GP.^35^ Moreover, the lowered lag of GP may be related to the ability of ruminal microbes to utilize plant secondary metabolites in turmeric rhizomes as an energy source to attach and degrade cell walls in the incubated diet.^6^ Salem et al.^36^ observed a decreased lag of GP with the administration of secondary metabolites in *Salix babylonica* extract.

The linear responses indicate that the phytochemical constituents in all the levels were within the acceptable ranges of secondary metabolites that did not impair the activities of the ruminal microbes. Appropriate levels of phytochemicals favorably increase GP.^9^ As previously noted, the levels of turmeric in the present experiment varied from 0.5 to 2% and were less than that reported by Aderinboye and Olanipekun^17^ who stated that turmeric inclusion level above 10 mg/g DM was detrimental to ruminal fermentation.

### 4.3. Methane production

Under normal feeding conditions, ruminal gases consist mainly of CO_2_, CH_4_, N and traces of O_2_.^37^ In case of any changes in the produced gas amount, normally, all individual gases would also change with the same scenario at the same time, keeping a semifixed proportion for individual gases. However, some dietary treatments/additives cause qualitative changes in the produced gases to increase/decrease one or more of them at the expense of other gases such as decreasing CH_4_ and increasing CO_2_.^38^^,^^39^ Therefore, calculating the proportion of produced gases is an important issue.^29^ In the present experiment, turmeric rhizomes at all levels decreased CH_4_ production and its percentage from the total gases. Kholif et al.^29^ observed that administration of fennel seeds decreased *in vitro* CH_4_ production when administered at 0.5 to 2% (DM basis). Tian et al.^16^ showed that feeding curcumin to lambs at 300 mg/g DM of diet lowered the number of methanogens and protozoans. Decreasing protozoa decreases CH_4_ production by 9–40%.^40^ Moreover, Aderinboye and Olanipekun^17^ observed lowered *in vitro* CH_4_ production with turmeric inclusion. Such results confirm the possibility of using phytochemicals as a sustainable approach to mitigate CH_4_ production from ruminants. Generally, phytogenic feed additives may reduce CH_4_ production due to the ability of phytochemicals to inhibit methanogens’ activity and decrease ruminal CH_4_ production.^41^ Moreover, phenolic compounds decrease CH_4_ production by inhibiting methanogen growth and reducing interspecies hydrogen transfer.^34^^,^^41^ Hydrogen not used for CH_4_ production is used in the synthesis of SCFA.^42^ In the rumen, H_2_ is produced from carbohydrates and amino acids fermentation and can be in two forms: dissolved or gas. The dissolved H_2_ is used by methanogens as a major energy source and later converted to CH_4_, but gas H_2_ is not used and only the dissolved H_2_ is biologically available for microbial growth. The rest of the H_2_ escapes from the rumen in a liquid phase.^43^

Turmeric rhizomes increased the lag of CH_4_ production, in consonance with other experiments where the administration of phytogenic fennel seeds increased the lag of CH_4_ production when administered at 0.5–2% (DM basis).^29^ The delayed onset of CH_4_ production suggests that the administration of turmeric rhizomes delayed methanogenic archaea and bacteria’s adaptation to the diet.

### 4.4. Degradability and fermentation

The sensitivity of ruminal fermentation to the feeding of phytochemicals is well documented.^6^^,^^12^ The pH values (6.10–6.27) were above the minimum value (5.6) for ruminal fiber degrading microbial activities and growth.^44^ Turmeric at 0.5 and 1% decreased ruminal pH, coinciding with the increase in total SCFA concentration. The lowered ruminal pH may be due to the enhanced nutrient digestibility, fermentation rate and SCFA production with turmeric inclusion.^45^^,^^46^

Unexpectedly, turmeric rhizomes’ administration increased ruminal NH_3_-N concentration. The phytochemicals (e.g., curcumin and turmerone) are well documented to increase the number of protein degrading bacteria (e.g. *Prevotella ruminicola*), and the activity of protease,^16^ which may explain the increased ruminal NH_3_-N concentration. Turmeric can reduce “hyper NH_3_-producing bacteria” and protein degradation or decrease amino acid deamination.^6^ Level of administration and incubated substrate may be the main reason for the increases because the effect of phytogenic compounds could affect ruminal N metabolism in a dose-dependent manner.^47^ High levels of plant secondary metabolites significantly reduced NH_3_-N in *vitro* batch cultures, whereas the low concentration of plant secondary metabolites increased NH_3_-N concentration.^47^ Tian et al.^16^ showed that feeding curcumin at 300 mg/kg diet to lambs lowered ruminal NH_3_-N, while Kholif et al.^29^ observed unaffected *in vitro* ruminal NH_3_-N concentration with the administration of fennel seeds. Interestingly, values (10.43–13.03 mg/dL) were within the normal range (8.5 to over 30 mg ammonia-N/dL) for optimal microbial growth and activity.^48^ More experiments with different levels of the main components in turmeric (e.g. curcumin and turmerone) are recommended to evaluate their effects on ruminal NH_3_-N production.^16^

The increased concentrations of total SCFA, acetate and propionate with turmeric inclusion indicate improved ruminal fermentation. Tian et al.^16^ showed that feeding curcumin at 300 mg/kg diet to lambs lowered ruminal pH and increased total SCFA and acetate concentrations. The present results may be attributed to increased carbohydrate fermentation by ruminal microorganisms.^16^ In the current experiment, the inclusion of turmeric rhizomes improved the DM and fiber degradabilities, which may explain the results of total and individual SCFA. Phytochemicals induce ruminal bacteria to degrade structural and nonstructural carbohydrates to produce acetate and propionate, respectively. Phytoconstituents in turmeric rhizomes may interact with microbial cell membranes and inhibit the growth of some gram-positive and gram-negative bacteria, resulting in higher propionate concentrations.^6^ The improved diet fermentability with turmeric inclusion produced more SCFA because SCFA is the main endproduct of ruminal carbohydrate fermentation. Improved fiber digestibility is beneficial since this is one of the main goals of ruminal modification.^49^ The increased acetate and propionate with the administration of turmeric culminated in unaffected acetate acid-to-propionate acid ratios. The ruminal acetate acid-to-propionate acid ratio is related to diet utilization efficiency.^50^ However, Lin et al.^50^ showed that the main factors affecting ruminal acetate acid-to-propionate acid ratio are the degradation rate of the diet and the rumen microbial structure, and the ratio is not related to the concentration of the fermentation substrate but is affected by the structure of the rumen microbiota.

In the present experiment, turmeric rhizomes increased the degradabilities of DM, NDF and ADF. Hodjatpanah-Montazeri et al.^34^ showed that the inclusion of turmeric extract at 40 and 80 mg/L of culture fluid increased *in vitro* DM degradability after 96 h of incubation. Similar results were observed by Kholif et al.^29^ when fennel seeds were included at 0.5–2% of incubated substrate (DM basis). These results may be related to the ability of plant phytochemicals (e.g., in turmeric rhizomes) to stimulate ruminal fibrolytic microbes’ activities and growth,^51^ resulting in a faster degradation rate and extent of substrates.^6^ As shown from the GC analysis of volatile compounds in turmeric rhizomes, the presence of phytochemicals and phenolics in the rhizomes may be considered the main reason due to their ability to stimulate microbial growth and activity within the rumen to degrade cell-wall constituents.^6^ Plant phytochemicals have been reported to stimulate fibrolytic microbial activities in the rumen^52^ which increase the rate of fermentation and degradation of substrates.^6^ Rumen microbiota can use plant phytochemicals (e.g., phenolics and essential oils) as energy sources for their growth and activity.^6^ Moreover, the antiprotozoal effects of the phytochemicals may be another reason.^6^^,^^16^ Tian et al.^16^ showed that feeding curcumin to lambs at 300 mg/g DM of the diet increased the activities of pectinase, carboxymethyl cellulose and protease, and the count of total bacteria, *Ruminococcus albus*, *Fibrobacter succinogenes* and *Prevotella ruminicola*.

Turmeric rhizomes increased ME, PF_24_ and MCP, indicating improved synchronization between energy and protein release in the rumen. The calculated values of ME were lower than those observed in feeds commonly used in ruminant diets. Abaş et al.^53^ showed that the ME of feeds ranged from 3.07 to 10.22 MJ/kg DM. The values of PF_24_ were higher than those of common feeds. The PF_24_ values are within a theoretical range (3.1–16.1) defined by Vercoe et al.^54^ Higher PF_24_ indicates incorporation of more degraded substrate into microbial mass (i.e., improved efficiency of MCP production).^54^ The high PF_24_ could be due to the solubilization of secondary metabolites (e.g., tannins) from the feed. These secondary metabolites would make no contribution to gas or energy in the system but would contribute to DM loss but not to GP.^54^ Hodjatpanah-Montazeri et al.^34^ observed increased PF_24_ when turmeric extract was included at 40 and 80 mg/L of culture fluid. Phytochemicals in the rhizomes of turmeric may interact with the biosynthesis of aromatic amino acids, as both biosynthesis pathways are linked through phytochemicals in the rhizomes.^55^ The results of MCP parallel with those of ruminal NH_3_-N, where increased concentrations of NH_3_-N provide ruminal microorganisms with N required for MCP synthesis.^56^

## 5. Conclusions

Administration of turmeric rhizomes appears to be a sustainable approach to mitigate methane production and improve dietary energy and protein utilization. Administration of turmeric rhizomes at 0.5–2% of the diet could potentially improve rumen fermentation kinetics, and increase gas production, ruminal ammonia-N and nutrient degradability. Additional research should concentrate on studying the effect of turmeric administration *in vivo* to further elucidate its effect on methane production and diet digestibility.

## Data Availability

The datasets used and/or analyzed during the current study are available from the corresponding author on reasonable request.

## References

[1] Low CX, Tan LT-H, Ab Mutalib N-S, et al. Unveiling the impact of antibiotics and alternative methods for animal husbandry: a review. *Antibiotics (Basel)*. 2021;10(5):578. 34068272 10.3390/antibiotics10050578 PMC8153128

[2] Khattab MSA, Kholif AE, Abd El Tawab AM, et al. Effect of replacement of antibiotics with thyme and celery seed mixture on the feed intake and digestion, ruminal fermentation, blood chemistry, and milk lactation of lactating Barki ewes. *Food Funct*. 2020;11(8):6889–12. 32691032 10.1039/d0fo00807a

[3] Hegazy SAA, Elmawla SM, Khorshed MM, Salem FA. Productive and immunological performance of small ruminants offered some medicinal plants as feed additives. *Int J Vet Sci*. 2022;12(1, 2023):120–125.

[4] Kholif AE. A review of effect of saponins on ruminal fermentation, health and performance of ruminants. *Vet Sci*. 2023;10(7):450. 37505855 10.3390/vetsci10070450 PMC10385484

[5] Vasta V, Daghio M, Cappucci A, et al. Invited review: Plant polyphenols and rumen microbiota responsible for fatty acid biohydrogenation, fiber digestion, and methane emission: Experimental evidence and methodological approaches. *J Dairy Sci*. 2019;102(5):3781–3804. 30904293 10.3168/jds.2018-14985

[6] Kholif AE, Olafadehan OA. Essential oils and phytogenic feed additives in ruminant diet: chemistry, ruminal microbiota and fermentation, feed utilization and productive performance. *Phytochem Rev*. 2021;20(6):1087–1108.

[7] Morsy TA, Kholif AE, Matloup OH, Elella AA, Anele UY, Caton JS. Mustard and cumin seeds improve feed utilisation, milk production and milk fatty acids of Damascus goats. *J Dairy Res*. 2018;85(2):142–151. 29478424 10.1017/S0022029918000043

[8] Kholif AE, Gouda GA, Galyean ML, Anele UY, Morsy TA. Extract of *Moringa oleifera* leaves increases milk production and enhances milk fatty acid profile of Nubian goats. *Agroforest Syst*. 2019;93(5):1877–1886.

[9] Salem AZM, Kholif AE, Elghandour MMY, Hernandez SR, Domínguez-Vara IA, Mellado M. Effect of increasing levels of seven tree species extracts added to a high concentrate diet on in vitro rumen gas output. *Anim Sci J*. 2014;85(9):853–860. 24796241 10.1111/asj.12218

[10] Ebeid HM, Mengwei L, Kholif AE, et al. *Moringa oleifera* oil modulates rumen microflora to mediate in vitro fermentation kinetics and methanogenesis in total mix rations. *Curr Microbiol*. 2020;77(7):1271–1282. 32130505 10.1007/s00284-020-01935-2

[11] Morsy TA, Gouda GA, Kholif AE. In vitro fermentation and production of methane and carbon dioxide from rations containing *Moringa oleifera* leave silage as a replacement of soybean meal: in vitro assessment. *Environ Sci Pollut Res*. 2022;29(46):69743–69752. 10.1007/s11356-022-20622-2 PMC9512743 35570255

[12] El-Zaiat HM, Kholif AE, Moharam MS, Attia MF, Abdalla AL, Sallam SMA. The ability of tanniniferous legumes to reduce methane production and enhance feed utilization in Barki rams: in vitro and in vivo evaluation. *Small Rumin Res*. 2020;193:106259.

[13] Zhang Q, Wu S, Zou X, et al. Effects of *Neolamarckia cadamba* leaves extract on methanogenesis, microbial community in the rumen and digestibility of stylo silage. *J Clean Prod*. 2022;369(23):133338.

[14] Orzuna-Orzuna J, Dorantes-Iturbide G, Lara-Bueno A, Mendoza-Martínez G, Miranda-Romero L, Hernández-García P. Effects of dietary tannins’ supplementation on growth performance, rumen fermentation, and enteric methane emissions in beef cattle: a meta-analysis. *Sustainability*. 2021;13(13):7410.

[15] Jafari S, Ebrahimi M, Goh YM, Rajion MA, Jahromi MF, Al-Jumaili WS. Manipulation of rumen fermentation and methane gas production by plant secondary metabolites (saponin, tannin and essential oil): a review of ten-year studies. *Ann Anim Sci*. 2019;19(1):3–29.

[16] Tian G, Zhang X, Hao X, Zhang J. Effects of curcumin on growth performance, ruminal fermentation, rumen microbial protein synthesis, and serum antioxidant capacity in housed growing lambs. *Animals (Basel)*. 2023;13(9):1439. 37174476 10.3390/ani13091439 PMC10177206

[17] Aderinboye RY, Olanipekun AO. An in-vitro evaluation of the potentials of turmeric as phytogenic feed additive for rumen modification. *NJAP*. 2021;48(3):193–203.

[18] Qin DM, Wang XB, Zou N, Han C, Xu J. Gas chromatography-mass spectrometry (GC-MS) analysis of the volatile oil of *Cichorium glandulosum* Boiss et huet and its effects on carbon tetrachloride-induced liver fibrosis in rats. *Med Sci Monit*. 2019;25:3591–3604. 31089070 10.12659/MSM.913445 PMC6532557

[19] Goering HK, Van Soest PJ. *Forage Fiber Analyses*. ARS-USDA; 1975.

[20] AOAC. *Official Methods of Analysis of AOAC International*. 16th ed. AOAC International; 1997.

[21] Abdel-Nasser A, Hathout AS, Badr AN, Barakat OS, Fathy HM. Extraction and characterization of bioactive secondary metabolites from lactic acid bacteria and evaluating their antifungal and antiaflatoxigenic activity. *Biotechnol Rep (Amst)*. 2023;38:e00799. 37206916 10.1016/j.btre.2023.e00799 PMC10189384

[22] Van Soest PJ, Robertson JB, Lewis BA. Methods for dietary fiber, neutral detergent fiber, and nonstarch polysaccharides in relation to animal nutrition. *J Dairy Sci*. 1991;74(10):3583–3597. 1660498 10.3168/jds.S0022-0302(91)78551-2

[23] NRC. *Nutrient Requirements of Dairy Cattle*. 7th ed. National Academies Press; 2001. 38386771

[24] France J, Dijkstra J, Dhanoa MS, Lopez S, Bannink A. Estimating the extent of degradation of ruminant feeds from a description of their gas production profiles observed in vitro: Derivation of models and other mathematical considerations. *Br J Nutr*. 2000;83(2):143–150. 10743493 10.1017/s0007114500000180

[25] Blümmel M, Steingass H, Becker K. The relationship between in vitro gas production, in vitro microbial biomass yield and ^15^N incorporation and its implications for the prediction of voluntary feed intake of roughages. *Br J Nutr*. 1997;77(6):911–921. 9227188 10.1079/bjn19970089

[26] Menke KH, Raab L, Salewski A, Steingass H, Fritz D, Schneider W. The estimation of the digestibility and metabolizable energy content of ruminant feeding stuffs from the gas production when they are incubated with rumen liquor in vitro. *J Agric Sci*. 1979;93(1):217–222.

[27] Gounder DK, Lingamallu J. Comparison of chemical composition and antioxidant potential of volatile oil from fresh, dried and cured turmeric (*Curcuma longa*) rhizomes. *Ind Crops Prod*. 2012;38(1):124–131.

[28] Poudel DK, Ojha PK, Rokaya A, Satyal R, Satyal P, Setzer WN. Analysis of volatile constituents in curcuma species, *viz. C. aeruginosa, C. zedoaria,* and *C. longa*, from Nepal. *Plants (Basel)*. 2022;11(15):1932. 35893636 10.3390/plants11151932 PMC9332366

[29] Kholif AE, Gouda GA, Fahmy M, Morsy TA, Abdelsattar MM, Vargas-Bello-Pérez E. Fennel seeds dietary inclusion as a sustainable approach to reduce methane production and improve nutrient utilization and ruminal fermentation. *Anim Sci J*. 2024;95(1):e13910. 38221575 10.1111/asj.13910

[30] Patra AK, Kamra DN, Agarwal N. Effects of extracts of spices on rumen methanogenesis, enzyme activities and fermentation of feeds in vitro. *J Sci Food Agric*. 2010;90(3):511–520. 20355074 10.1002/jsfa.3849

[31] Hassan F-U, Arshad MA, Ebeid HM, et al. Phytogenic additives can modulate rumen microbiome to mediate fermentation kinetics and methanogenesis through exploiting diet–microbe interaction. *Front Vet Sci*. 2020;7:575801. 33263013 10.3389/fvets.2020.575801 PMC7688522

[32] Kholif AE, Hassan AA, El Ashry GM, et al. Phytogenic feed additives mixture enhances the lactational performance, feed utilization and ruminal fermentation of Friesian cows. *Anim Biotechnol*. 2021;32(6):708–718. 32248772 10.1080/10495398.2020.1746322

[33] Kholif AE, Hassan AA, Matloup OH, El Ashry GM. Top-dressing of chelated phytogenic feed additives in the diet of lactating Friesian cows to enhance feed utilization and lactational performance. *Ann Anim Sci*. 2021;21(2):657–673.

[34] Hodjatpanah-Montazeri A, Mesgaran MD, Vakili A, Ghorbani B, Tabatabaie F. In vitro effect of garlic oil and turmeric extract on methane production from gas test medium. *ARRB*. 2014;4(9):1439–1447.

[35] Elghandour MMY, Kholif AE, Bastida AZ, Martínez DLP, Salem AZM. In vitro gas production of five rations of different maize silage and concentrate ratios influenced by increasing levels of chemically characterized extract of *Salix babylonica*. *Turk J Vet Anim Sci*. 2015;39(2):186–194.

[36] Salem AZM, Kholif AE, Olivares M, Elghandour MMY, Mellado M, Arece J. Influence of *S. babylonica* extract on feed intake, growth performance and diet in vitro gas production profile in young lambs. *Trop Anim Health Prod*. 2014;46(1):213–219. 24077921 10.1007/s11250-013-0478-0

[37] Wang Y, Wang L, Wang Z, et al. Recent advances in research in the rumen bloat of ruminant animals fed high-concentrate diets. *Front Vet Sci*. 2023;10:1142965. 37035805 10.3389/fvets.2023.1142965 PMC10076780

[38] Elghandour MMY, Kholif AE, Salem AZM, Olafadehan OA, Kholif AM. Sustainable anaerobic rumen methane and carbon dioxide productions from prickly pear cactus flour by organic acid salts addition. *J Clean Prod*. 2016;139:1362–1369.

[39] Elghandour MMY, Kholif AE, Salem AZM, et al. Addressing sustainable ruminal methane and carbon dioxide emissions of soybean hulls by organic acid salts. *J Clean Prod*. 2016;135:194–200.

[40] Dohme F, Machmuller A, Estermann BL, Pfister P, Wasserfallen A, Kreuzer M. The role of the rumen ciliate protozoa for methane suppression caused by coconut oil. *Lett Appl Microbiol*. 1999;29(3):187–192.

[41] Ku-Vera JC, Jiménez-Ocampo R, Valencia-Salazar SS, et al. Role of secondary plant metabolites on enteric methane mitigation in ruminants. *Front Vet Sci*. 2020;7:584. 33195495 10.3389/fvets.2020.00584 PMC7481446

[42] Ungerfeld EM. Metabolic hydrogen flows in rumen fermentation: principles and possibilities of interventions. *Front Microbiol*. 2020;11:589. 32351469 10.3389/fmicb.2020.00589 PMC7174568

[43] Wang M, Sun XZ, Janssen PH, Tang SX, Tan ZL. Responses of methane production and fermentation pathways to the increased dissolved hydrogen concentration generated by eight substrates in in vitro ruminal cultures. *Anim Feed Sci Technol*. 2014;194:1–11.

[44] Ryle M, Ørskov ER. *Energy Nutrition in Ruminants*. Netherlands: Springer; 1990.

[45] Mahmoud AEM, Rahmy HAF, Ghoneem WMA. Role of caraway, fennel and melissa addition on productive performance of lactating Frisian cows. *Pak J Biol Sci*. 2020;23(11):1380–1389. 33274865 10.3923/pjbs.2020.1380.1389

[46] Moosavi-Zadeh E, Rahimi A, Rafiee H, Saberipour H, Bahadoran R. Effects of fennel (*Foeniculum vulgare*) seed powder addition during early lactation on performance, milk fatty acid profile, and rumen fermentation parameters of Holstein cows. *Front Anim Sci*. 2023;4(2)

[47] Cardozo PW, Calsamiglia S, Ferret A, Kamel C. Screening for the effects of natural plant extracts at different pH on in vitro rumen microbial fermentation of a high-concentrate diet for beef cattle. *J Anim Sci*. 2005;83(11):2572–2579. 16230654 10.2527/2005.83112572x

[48] Jones M, Jones G. *Animal Nutrition*. 7th ed. Pearson Education Limited; 2012.

[49] Jouany JP, Morgavi DP. Use of “natural” products as alternatives to antibiotic feed additives in ruminant production. *Animal*. 2007;1(10):1443–1466. 22444918 10.1017/S1751731107000742

[50] Lin X, Hu Z, Zhang S, et al. A study on the mechanism regulating acetate to propionate ratio in rumen fermentation by dietary carbohydrate type. *ABB*. 2020;11(08):369–390.

[51] Singla A, Hundal JS, Patra AK, Wadhwa M, Nagarajappa V, Malhotra P. Effect of dietary supplementation of *Emblica officinalis* fruit pomace on methane emission, ruminal fermentation, nutrient utilization, and milk production performance in buffaloes. *Environ Sci Pollut Res Int*. 2021;28(14):18120–18133. 33405166 10.1007/s11356-020-12008-z

[52] Morgavi DP, Newbold CJ, Beever DE, Wallace RJ. Stability and stabilization of potential feed additive enzymes in rumen fluid. *Enzyme Microb Technol*. 2000;26(2-4):171–177. 10689074 10.1016/s0141-0229(99)00133-7

[53] Abaş I, Özpinar H, Kutay HC, Kahraman R, Eseceli H. Determination of the metabolizable energy (ME) and net energy lactation (NEL) contents of some feeds in the Marmara Region by in vitro gas technique. *Turk J Vet Anim Sci*. 2005;29(3):751–757.

[54] Vercoe PE, Makkar HPSS, Schlink AC. *In Vitro Screening of Plant Resources for Extra-Nutritional Attributes in Ruminants: Nuclear and Related Methodologies*. Netherlands: Springer; 2010.

[55] Mandal SM, Chakraborty D, Dey S. Phenolic acids act as signaling molecules in plant-microbe symbioses. *Plant Signal Behav*. 2010;5(4):359–368. 20400851 10.4161/psb.5.4.10871 PMC2958585

[56] Boucher SE, Ordway RS, Whitehouse NL, Lundy FP, Kononoff PJ, Schwab CG. Effect of incremental urea supplementation of a conventional corn silage-based diet on ruminal ammonia concentration and synthesis of microbial protein. *J Dairy Sci*. 2007;90(12):5619–5633. 18024754 10.3168/jds.2007-0012

